# Investigating the role of interleukin-1 beta and glutamate in inflammatory bowel disease and epilepsy using discovery browsing

**DOI:** 10.1186/s13326-018-0192-y

**Published:** 2018-12-27

**Authors:** Thomas C. Rindflesch, Catherine L. Blake, Michael J. Cairelli, Marcelo Fiszman, Caroline J. Zeiss, Halil Kilicoglu

**Affiliations:** 1 Retired, Washington, DC, USA; 2 School of Information Sciences, University of Illinois at Urbana-Champaign, 501 E Daniel Street, Champaign, 61820 IL USA; 3 Kaiser Permanente Southern California, 11975 El Camino Real, San Diego, CA, 92103 USA; 4 Independent researcher, Rio de Janeiro, Brazil; 5 Department of Comparative Medicine, Yale School of Medicine, New Haven, CT, 06520 USA; 6 Lister Hill National Center for Biomedical Communications, U.S. National Library of Medicine, 8600 Rockville Pike, Bethesda, MD, USA

**Keywords:** Literature-based discovery, Discovery browsing, Epilepsy, Inflammatory bowel disease, Interleukin-1 beta, Glutamate

## Abstract

**Background:**

Structured electronic health records are a rich resource for identifying novel correlations, such as co-morbidities and adverse drug reactions. For drug development and better understanding of biomedical phenomena, such correlations need to be supported by viable hypotheses about the mechanisms involved, which can then form the basis of experimental investigations.

**Methods:**

In this study, we demonstrate the use of discovery browsing, a literature-based discovery method, to generate plausible hypotheses elucidating correlations identified from structured clinical data. The method is supported by Semantic MEDLINE web application, which pinpoints interesting concepts and relevant MEDLINE citations, which are used to build a coherent hypothesis.

**Results:**

Discovery browsing revealed a plausible explanation for the correlation between epilepsy and inflammatory bowel disease that was found in an earlier population study. The generated hypothesis involves interleukin-1 beta (IL-1 beta) and glutamate, and suggests that IL-1 beta influence on glutamate levels is involved in the etiology of both epilepsy and inflammatory bowel disease.

**Conclusions:**

The approach presented in this paper can supplement population-based correlation studies by enabling the scientist to identify literature that may justify the novel patterns identified in such studies and can underpin basic biomedical research that can lead to improved treatments and better healthcare outcomes.

## Background

Information needs in clinical setting and basic research setting differ significantly. Studies of information needs in clinical setting have focused on the types of questions asked by clinicians [1] and have informed the specific information facets (population, intervention, comparison, and outcome) that need to be identified in order to address those clinical questions [2]. In contrast to the clinical setting where there are often multiple studies relevant to the clinical encounter, scientists operate at the discovery end of the information synthesis spectrum where there is less information available, and agreement on how to evaluate or combine findings from different studies is still under development [3]. In such an environment, a scientist begins with what is best described as a hypothesis projection, “the purely conjectural proliferation of a whole gamut of alternative explanatory hypotheses that are relatively plausible, a proliferation based on guesswork - though not ’mere’ guesswork, but guesswork guided by a scientifically trained intuition. The aim of this enterprise is to identify those hypotheses that merit detailed scrutiny.” [4]

Structured data from electronic health records (EHRs) are increasingly mined to identify novel correlations, such as disease co-occurrences or adverse drug reactions [5]. Such studies are sometimes highly localized, relying on data collected from a small set of institutions; thus, they can violate some of the key assumptions made when using traditional statistical measures to determine significance, leading to false positive associations [6]. When performed with population-level data (e.g., Medicare claims data), these data mining studies can provide epidemiological evidence for co-morbidities and other biomedical phenomena; however, they alone are unable to elucidate the mechanisms involved in such phenomena or offer plausible explanations. Such epidemiological evidence must be subjected to further analysis by scientists in order to generate viable hypotheses about the etiology of the observed correlations, a critical step for the development of safe and effective treatments.

In this paper, guided by statistical correlations extracted from structured EHR data, we show how a literature-based discovery technique called *discovery browsing* [7, 8] can be used to support scientists as they explore hypothesis projections. This study was instigated to generate a hypothesis of mechanism for the results of a recent retrospective population study that measured the relationship between epilepsy and twelve autoimmune diseases [9]. That study analyzed health insurance claims data for 2,518,034 patients, both male and female, 65 years or younger. They reported that the risk of epilepsy was significantly heightened among patients with autoimmune disease. Collectively, individuals with autoimmune disease accounted for 17.5% of patients with epilepsy in the study population. This was a significant result that was not generally anticipated in the clinical community. The authors made several suggestive observations relevant to this correlation. Glutamate receptors may be involved in the etiology of epilepsy and other central nervous system disorders (the glutamate hypothesis) [10–12]. The inflammatory component of autoimmune diseases may be responsible for the occurrence of epilepsy [13, 14]. They did not, however, propose possible mechanisms underlying their findings and observations. Because there exists a lack of mechanistic understanding for the relationship between epilepsy and autoimmune disease, we view it as a prime candidate for discovery browsing. The suggestive observations mentioned above combined with our prior work that investigated the relationship between major depression and inflammation via cytokines using discovery browsing [7] indicated to us that focusing on glutamate and inflammation could be a fruitful avenue for a mechanistic understanding.

In this study, we focus on epilepsy and one autoimmune disorder, inflammatory bowel disease (IBD, a broader term covering both Crohn’s disease and ulcerative colitis as included in Ong et al. [9]). Further, we look at interleukin-1 beta (IL-1 beta), as one of the principal substances (along with interleukin-6) involved in inflammation. We use discovery browsing to generate a hypothesis about the mechanisms of both IL-1 beta and glutamate, and suggest that the influence of the former on the latter is involved in the etiology of both IBD and epilepsy, thus proposing a mechanism for the observed connection between these two disorders. This study also investigates generalizability of previous work on discovery browsing. Instead of using discovery browsing to elucidate a general phenomenon that was observed in many different studies and known anecdotally for years (e.g., *obesity paradox* [8]), we apply it to a narrower scope, exploring possible mechanisms for the results of a single study without such an obvious presence in the clinical community.

## Related work

### Discovery browsing

Literature-based discovery (LBD) [15] is a method of hypothesis generation, the core premise of which is the so-called ABC paradigm. AB (a relationship between two terms A and B) and BC (a relationship between B and C) are both known, but an AC relationship has so far not been proposed. The method can be used for open discovery, in which the discovery (or hypothesis) AC is the result. Alternatively, in closed discovery, AC may be known (or assumed) and relations AB and BC are sought to posit B as an explanation for AC (or a mechanistic link between the two concepts). While LBD research has predominantly focused on biomedical literature, it has also been applied to other domains, such as humanities [16], world wide web [17], as well as technology and social issues [18].

Wilkowski et al. [7] introduced discovery browsing as a modification of LBD. They described it as a tool for illuminating under-studied and poorly understood phenomena rather than necessarily for making discoveries. Discovery browsing also relies on the ABC paradigm and the relationships it exhibits; however, the researcher may assume (or already know) the relationships, but seek to elucidate the details of these assumptions, hypotheses, or known relationships. In the current study, we consider the relationships to be IBD (A) – inflammation (B) – epilepsy (C), in which AB, BC, and AC have all been proposed. We then seek to investigate, expand, and elucidate the B relationship between these two diseases for a finer-grained understanding of the mechanisms involved.

Wilkowski et al. [7] used discovery browsing to look at the interaction of melatonin, cytokines, and major depression. Cairelli et al. [8] formalized the method and exploited it to investigate why obesity is beneficial in intensive care, but detrimental otherwise (i.e. *obesity paradox*).

### SemRep

Semantic predications extracted from MEDLINE citations (titles and abstracts) naturally correlate with relations in the ABC paradigm and underpin this study. A semantic predication is a formal representation of an assertion made in text. Such structures provide a type of computable knowledge representing the information in the text from which they are extracted. For example, the predication “Interleukin-1 beta-CAUSES-Seizures” represents part of the meaning of the sentence, “*In addition, high* ***IL-1beta*** doses ***induced*** ***seizures*** *only in IL-1beta receptor-expressing mice*” (mentions relevant to the predication in bold). Note that it does not (necessarily) summarize an entire sentence, and in this case does not contain the information limiting the seizure induction to IL-1 beta receptor-expressing mice. A semantic predication consists of a predicate (CAUSES in this example) and arguments (Interleukin-1 beta and Seizures).

We extract predications using the SemRep natural language processing system [19]. SemRep inspects each sentence of input text to identify predications asserted in each sentence. The system depends on domain knowledge in the Unified Medical Language System (UMLS) developed by the U.S. National Library of Medicine [20, 21]. A SemRep predication has UMLS Metathesaurus concepts as arguments and a UMLS Semantic Network relation as predicate.

Extracted predications may be filtered by using automatic abstraction summarization [22] to focus on specific aspects of biomedicine, such as treatment of diseases or pharmacogenomics. In this study, we used this process to focus on predications asserting core relations in molecular biology [23]. We used the following meta-predications, where the arguments are represented as general semantic classes:

- {Substance} ASSOCIATED_WITH OR PREDISPOSES OR CAUSES
  {Pathology}
- {Substance} INTERACTS_WITH OR INHIBITS OR STIMULATES
  {Substance}
- {Substance} AFFECTS OR DISRUPTS OR AUGMENTS {Anatomy OR Process}
- {Anatomy OR Living Being} LOCATION_OF {Substance}
- {Anatomy} PART_OF {Anatomy OR Living Being}
- {Process} PROCESS_OF {Living Being}

### Semantic MEDLINE

**Fig. 1:**
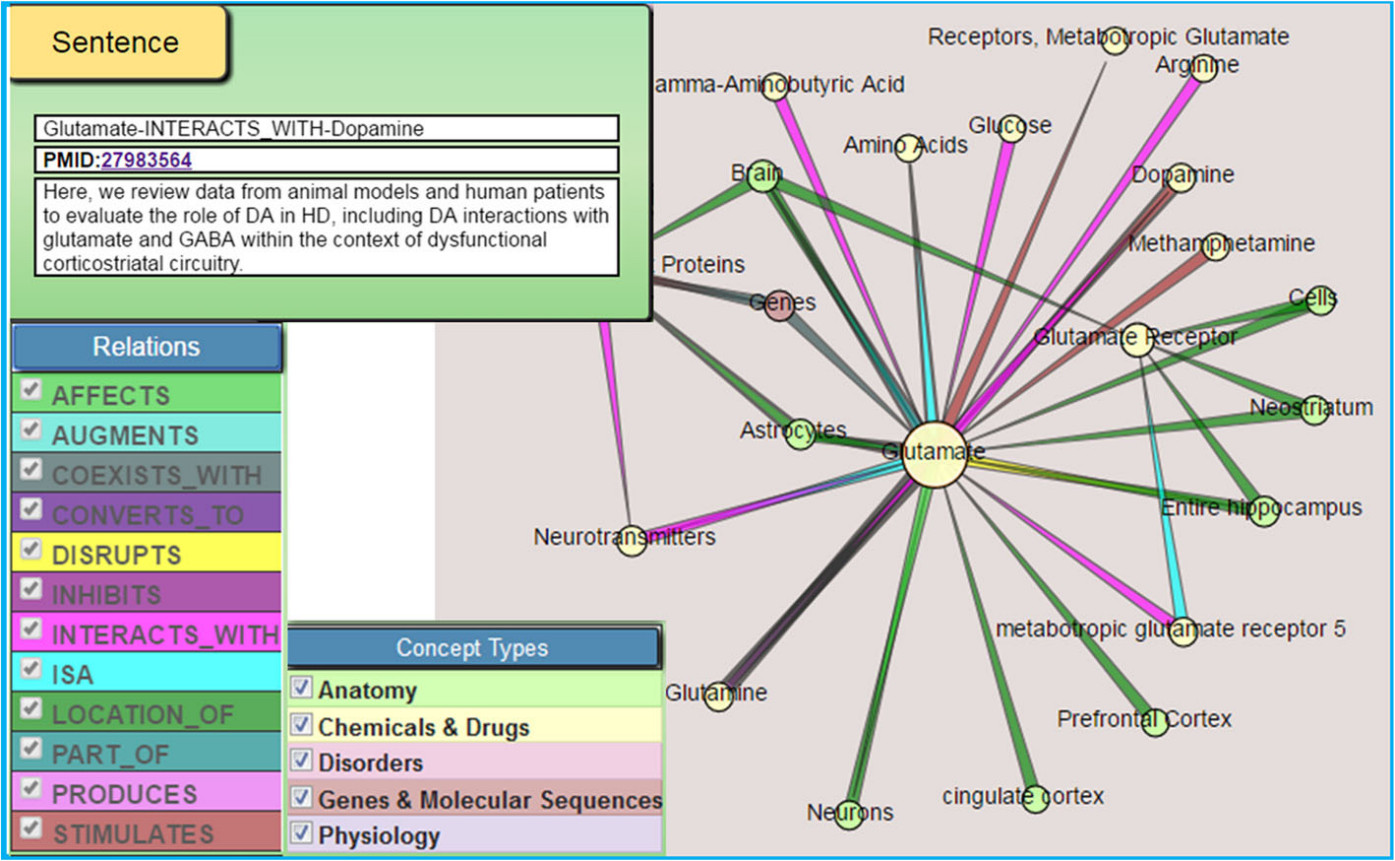
Illustration of the Semantic MEDLINE web application. The summarized results of a PubMed search are displayed as an interactive graph, where nodes represent subjects and objects of semantic predications and the edges represent the predicates (right). Edges are linked to the original MEDLINE citation from which the predication is extracted (top-left). Nodes and edges can be filtered using relation and semantic group filters (bottom-left)

The methodology pursued in this study is implemented with Semantic MEDLINE [24], a Web application that integrates PubMed document retrieval, SemRep natural language processing, automatic abstraction summarization, and visualization into a single Web portal. SemRep predications extracted from all MEDLINE citations are made available from SemMedDB [25] and are summarized according to the meta-predications just noted. Summarized predications are then presented as a connected interactive graph of semantic relations (Fig. 1). Subjects and objects are nodes in the graph, while predicates are edges. By clicking on an edge, the user can see the predication represented. The edge has a link to the original MEDLINE citation from which the predication is extracted.

Semantic MEDLINE supports discovery browsing by presenting relationships to the user that might not have been noticed without it. Miller et al. [26] used Semantic MEDLINE to study the effect of the interaction of testosterone and cortisol on declining sleep quality in aging men. In related work, Cohen et al. [27] discussed EpiphaNet, which displays SemRep predications in graphical form for literature-based discovery. Hristovski et al. [28] described several Semantic MEDLINE-based systems designed to facilitate discovery.

Other literature-based exploration and discovery systems have also been proposed to formulate and assess scientific hypotheses. For example, Arrowsmith [29] links two sets of articles from biomedical literature using title words and phrases and statistical information. In a similar vein, LitLinker [30] performs open discovery using UMLS concepts identified by MetaMap [31] as the basis, grouping and pruning them in conjunction with statistical correlations. Berlanga et al. [32] focus on semantic integration and visualization from multiple knowledge and data sources, using named entity recognition to recognize concepts, exploiting concept taxonomies and co-occurrence across documents to identify interesting associations and visualize them for exploration purposes. HyQue [33] is concerned with semantic integration for the purpose of hypothesis evaluation and uses Semantic Web technologies to standardize representation of input, knowledge sources, data, queries, and outputs. Unlike SemRep, these systems/tools do not perform explicit relation extraction, mainly relying on concept co-occurrence or manually curated relationships.

### Inflammation, epilepsy, and inflammatory bowel disease

Before investigating possible mechanistic connections between IBD and epilepsy, we surveyed MEDLINE regarding the observations from Ong et al. [9] to determine how much research has been published concerning this relationship. We began by looking at IBD, a disorder in which the involvement of inflammation is not controversial.

In order to focus relevant background information, we used Semantic MEDLINE as an adjunct to PubMed to query MEDLINE for the primary proinflammatory cytokines involved in IBD. We issued the Semantic MEDLINE query “inflammatory bowel disease” on 03/22/2017 and restricted results to the most recent 500 citations. 4402 predications were extracted and we restricted these to the core relations in molecular biology. The following cytokines appear as nodes in the graph: IL-1 alpha, IL-1 beta, IL-6, IL-10, IL-17, IL-18, IL-19, and IL-23. We then used PubMed to determine the amount of research for each of these substances in association with IBD. In order to achieve high recall, we combined the search for IL-1 alpha and IL-1 beta with the query (“interleukin-1” AND “inflammatory bowel disease”). All other cytokines in this list were queried with the form (“ <IL-X >” AND “inflammatory bowel disease”). The query results were: IL-1: 340 MEDLINE citations; IL-6: 779; IL-10: 841; 1L-17: 348; IL-18: 106; IL-19: 10; IL-23: 228.

In order to determine which cytokines are most prominently involved in both IBD and epilepsy, we then repeated this series of queries to PubMed, substituting “epilepsy” for “inflammatory bowel disease”. The results for each query were: IL-1: 201 MEDLINE citations; IL-6: 216; IL-10: 71; IL-17: 19; IL-18: 14; IL-23: 4; and no citations retrieved for IL-19. Although several cytokines are prominent in IBD research, only IL-1 (mostly beta) and interleukin-6 have been much studied with respect to epilepsy. The inflammatory aspects of both conditions are accompanied by alterations in a broad array of mediators (see Bevivino and Monteleone [34] and Matin et al. [35] for reviews on IBD and epilepsy, respectively) whose interaction in disease etiology is likely to be contextual. While both IL-1 beta and interleukin-6 were promising candidates for discovery browsing, we focused on IL-1 beta in this paper, partly to keep the scope of this work manageable. This procedure could be repeated with other substances, especially interleukin-6, to potentially reveal additional insights.

As further background investigation, we used PubMed to get an overview of research on IL-1 beta and IBD. We looked at a sample from the 340 citations returned with the query noted above. IL-1 beta has long been associated with gastrointestinal disturbances (e.g. [36]) and with IBD in particular (e.g. [37]). Subsequent research has looked at various aspects of that association. For example, Casellas et al. [38] investigated the role of IL-1 beta in chronic ulcerative colitis. Heresbach et al. [39] sought to elucidate genetic susceptibility to IBD, concentrating on IL-1beta and IL-1 receptor antagonist (IL-1ra) gene polymorphisms. Coccia et al. [40] reported on multiple mechanisms through which IL-1 beta contributes to intestinal pathology. Li et al. [41] exploited bioluminescence imaging to determine the location of cells producing IL-1 beta during intestinal inflammation. Das [42] hypothesized that the etiology of IBD is due to inadequate production of inflammation resolving molecules, such as lipoxins, resolvins, protectins, maresins and nitrolipids.

Finally, we queried PubMed to determine whether there is any research on IL-1 beta and both IBD and epilepsy. The query (“interleukin 1” AND (“inflammatory bowel disease” OR colitis) AND (seizure OR epilepsy)) returned only 1 citation [43], a review which states in the abstract, foreshadowing the conclusions of Ong et al. [9], that “There are reports suggesting more predispositions to seizures during inflammatory conditions like colitis, pneumonia and rheumatoid arthritis.”

We then moved on to the focus of the paper, which was twofold: 1) investigate the research on IL-1 beta and epilepsy, and 2) look at possible mechanistic connections between IBD and epilepsy involving inflammation (IL-1 beta).

## Methods

**Fig. 2:**
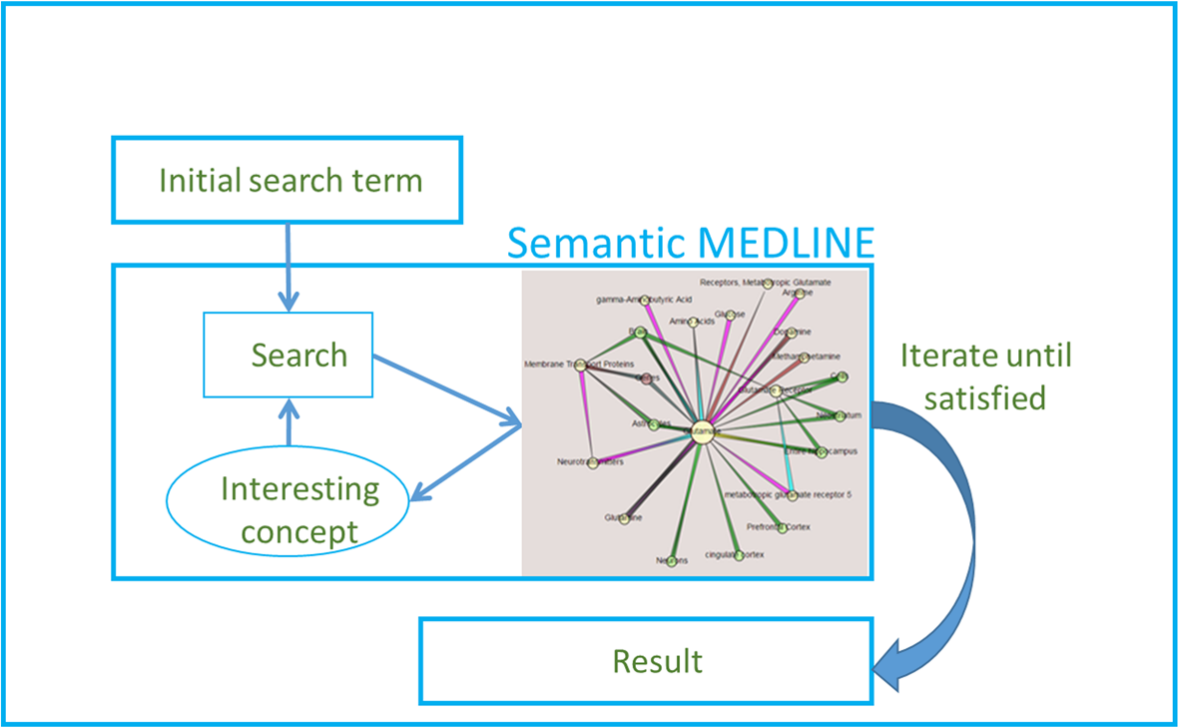
Overview of discovery browsing. An iterative process that incorporates Semantic MEDLINE help identify interesting concepts, which are used to build an argumentation chain

At the core of the discovery browsing methodology pursued in this study is *cooperative reciprocity* between the system and the user’s domain knowledge. This takes the form of the user issuing an initial query to Semantic MEDLINE reflecting an area of interest. All queries were issued at the end of March, 2017. The graph resulting from each query was inspected for concepts (either in the predications or in the abstracts from which they are extracted) that capture the attention of the researcher and which may incite the development of a potential hypothesis regarding the study being pursued. At this point, PubMed was consulted (using the same query) to determine whether any citation from which Semantic MEDLINE did not extract a predication supported the viability of the hypothesis being developed. This step is performed in part to address recall problems of SemRep, which may result in missing information important for hypothesis generation. If the developing hypothesis was supported, it was pursued with another query to Semantic MEDLINE incorporating the concept of interest, and the process was repeated until we were satisfied with a coherent argumentation chain. Finally, PubMed was searched to determine whether the hypothesis generated is novel. Figure 2 provides an overview of the method.

For each query to Semantic MEDLINE, we limited the abstracts returned to the most recent 500, although fewer total abstracts were retrieved for some queries. The predications extracted were then summarized using the meta-predications given in previous section. For ease of inspection, we further limited the graph for each query to a maximum of 50 nodes and 100 edges; nodes are ranked by frequency and the graph is limited to 50 nodes with the highest frequency.

## Results

### IL-1 beta and epilepsy

The initial query to Semantic MEDLINE was (“interleukin 1” AND (epilepsy OR seizure)), which returned 2481 predications extracted from 240 citations. In the summarized graph, several predications were considered noteworthy as indicating a relationship between IL-1 beta and epilepsy. “Interleukin-1 beta-AFFECTS-Seizure” was extracted from Vezzani et al. [44], which reports that intrahippocampal application of recombinant IL-1ra inhibits seizures experimentally induced by bicuculline methiodide in rodents. This study cites previous work [45], in which they found that exogenous application of IL-1 beta in the rat hippocampus prolongs kainite-induced seizure by enhancing glutamatergic neurotransmission.

The predication “Interleukin-1 beta-CAUSES-Seizures” was extracted from two citations. In one, Ravizza and Vezzani [46] conducted immunohistochemical analysis of tissue following acute electrical stimulation in the ventral hippocampus of rats. They investigated the role of IL-1 beta during resulting epileptic activity, focusing on the role of IL-1 receptor type 1 (IL-1R1) in rat forebrain. They suggest that this receptor plays different roles in neurons and in astrocytes during status epilepticus. Another study [47] looked at the mechanism of seizure by injecting the right lateral ventricle of rats with both IL-1 beta and glutamate. They conclude that there is an interaction between IL-1 beta (through the IL-1 receptor) and metabotropic glutamate receptors in the onset of epilepsy. “Interleukin-1 beta-AUGMENTS-Status Epilepticus” was extracted from Pernot et al. [48], in which the relationship between neuroinflammation and mesiotemporal lobe epilepsy syndrome was explored with immunohistochemical analysis of tissue after mesiotemporal lobe epilepsy syndrome was experimentally induced in C57BL/6 adult mice by the unilateral intrahippocampal injection of kainate. They conclude that neuroinflammatory pathways are associated with epileptogenesis.

Opposing results have also been published. One such study, Claycomb et al. [49] (from which the predication “Interleukin-1 beta-ASSOCIATED_WITH-Seizures” was extracted), reports that IL-1 beta is neuroprotective. This study was conducted on transgenic mice with targeted disruption in genes for either the ligand IL-1 beta or its signaling receptor, IL-lR1. Their claim is based on their finding that chemoconvulsants administered to IL-1 beta and IL-1R1 -/- mice produced more acute seizures than in their respective +/+ littermates. It is not clear that these results would generalize to animals without such genetic manipulation. See Table 1 for an overview of our results on IL-1 beta and epilepsy.

**Table 1:** Summary of articles discussing IL-1 beta and epilepsy

*Study*	*Subjects*	*Method*	*Result/Conclusion*
Vezzani et al. [45]	Kainite-intoxicated rats	Application of IL-1 beta in the hippocampus	IL-1 beta prolongs experimentally induced seizures
Vezzani et al. [44]	Bicuculline methiodide-intoxicated rodents	Intrahippocampal application of recombinant IL-1ra	IL-1ra inhibits experimentally induced seizures
Ravizza and Vezzani [46]	Male Sprague-Dawley rats	Immunohistochemical analysis following acute electrical stimulation in the ventral hippocampus	IL-1R1 plays different roles in neurons and in astrocytes during status epilepticus
Wang et al. [47]	Rats	Injection of right lateral ventricle with both IL-1 beta and glutamate	Interaction between IL-1 beta and metabotropic glutamate receptors in the onset of epilepsy
Pernot et al. [48]	C57BL/6 adult mice	Immunohistochemical analysis of tissue after mesiotemporal lobe epilepsy syndrome induced by intrahippocampal injection of kainate	Neuroinflammatory pathways are associated with epileptogenesis
Claycomb et al. [49]	IL-1 beta and IL1R1 -/- mice	Administration of chemoconvulsants	Produced more acute seizures

Since we saw considerable research implicating IL-1 beta in epileptogenesis, we next pursued the potential interaction of IL-1 beta and glutamate in the pathogenesis of epilepsy and seizures [45, 47]. We began by looking for research that examined glutamate and epilepsy without considering IL-1 beta, and then looked at the interaction of the two in epilepsy and seizure.

### Glutamate and epilepsy

The Semantic MEDLINE query (glutamate AND (epilepsy OR seizure)) extracted 5170 predications from the most recent 500 citations. After summarization, we examined several predications which appeared to be relevant to glutamate in the context of seizure or epilepsy. Juhasz et al. [50] used proton magnetic resonance spectroscopic imaging to test glutamate concentration levels in epileptic children with Sturge-Weber syndrome (which is strongly associated with epilepsy [51]). They found increased glutamate in the affected hemisphere, which they interpret as support for the role of excess glutamate in these patients (“Glutamate-ASSOCIATED_WITH-Seizures”). Cavus et al. [52] measured glutamate levels in epileptic and nonepileptic cortical sites in 79 patients with refractory epilepsy using high-performance liquid chromatography. They found elevated extracellular glutamate at epileptogenic as compared to nonepileptogenic sites (“Glutamate-ASSOCIATED_WITH-Epilepsy”).

In considering MEDLINE citations from which Semantic MEDLINE did not extract a predication, one notable paper discusses research on the mechanisms of glutamate involvement in epilepsy. Perez et al. [53] assume that excessive glutamate underlies refractory temporal lobe epilepsy. They investigated the cause by using immunogold electron microscopy to measure glutamate levels in tissue extracted from the brains of male Sprague-Dawley rats infused with methionine sulfoximine, which induces glutamine synthetase efficiency. They conclude that such deficiency leads to increased extracellular glutamate. The studies we report on glutamate and epilepsy are summarized in Table 2.

**Table 2:** Summary of articles discussing glutamate and epilepsy

*Study*	*Subjects*	*Method*	*Result/Conclusion*
Juhász et al. [50]	Epileptic children with Sturge-Weber syndrome	Proton magnetic resonance spectroscopic brain imaging	Increased glutamate concentrations observed
Cavus et al. [52]	Epileptic and nonepileptic cortical sites in patients with refractory epilepsy	High-performance liquid chromatography based on microdialysis probes	Elevated extracellular glutamate observed at epileptogenic sites
Perez et al. [53]	Tissue extracted from brains of male Sprague Dawley rats infused with methionine sulfoximine	Glutamate levels measured with immunogold electron microscopy	Glutamine synthetase deficiency leads to increased extracellular glutamate

Based on research indicating glutamate involvement in epilepsy and considering research implicating IL-1 beta in epilepsy, we were encouraged to investigate the interaction of IL-1 beta and glutamate in the context of this disorder.

### IL-1 beta and glutamate

We issued three queries to Semantic MEDLINE to investigate the relationship of IL-1 beta and glutamate in the etiology of epilepsy. One focused on this disorder (and seizure), another specified the brain (but not the disorder), and a third specified neither disorder nor anatomic location.

#### And epilepsy

The query (“interleukin-1” AND glutamate AND (seizure OR epilepsy)) retrieved 18 citations and 202 predications, which were not summarized. Two papers identified in the graph were relevant. Xiaoqin et al. [54] injected the cerebral cortex and hippocampus of rats with IL-1 beta and IL-6. Immunohistochemistry observation revealed the development of seizures along with increased glutamate and decreased GABA (“Interleukin-6-CAUSES-Seizures”). Donnelly et al. [55] analyzed synaptosomes prepared from the brains of BALB/c female mice, 8-12 weeks old, in which epilepsy-like symptoms had been induced with glycerol. Synaptosome pellets were then subjected to a series of in vitro techniques after which they observed an increase in IL-1 beta levels and a decrease in glutamate release in hippocampus tissue (“Entire hippocampus-LOCATION_OF-Glutamate”).

#### In the brain

The Semantic MEDLINE query (“interleukin 1” AND glutamate AND brain) returned 1850 predications from 160 citations. After summarization, several predications were extracted from citations discussing the interaction of IL-1 beta and glutamate. “Interleukin-1 beta-DISRUPTS-uptake” was extracted from an article [56], which reported that astrocyte uptake of glutamate is neuroprotective during brain inflammation. Based on Northern blot analysis and other in vitro techniques performed on primal human astrocyte cultures subjected to several cytokines and 3H-glutamate, the authors concluded that proinflammatory cytokines inhibit astrocyte glutamate uptake. Based on intracerebral microdialysis in unanesthetized rabbits, Huang et al. [57] reported that organum vasculosum laminae terminalis (OVLT) release of glutamate was induced by intracerebroventricular injection of IL-1beta (“Interleukin-1 beta-STIMULATES-Glutamate”).

When inspecting citations from which Semantic MEDLINE did not extract a predication, we found an earlier report which concluded that IL-1 beta enhances glutamate. As measured by brain microdialysis in freely moving male Sprague–Dawley rats, Mascarucci et al. [58] found that injection of intraperitoneal IL-1 beta increased glutamate release in the nucleus tractus solitarius.

Some studies reported that IL-1 beta inhibits glutamate. Murray et al. [59] prepared hippocampal synaptosomes from male Wistar rats, on which in vitro experiments were conducted. They reported that immunoblotting with specific antibody revealed that IL-1 beta inhibited potassium chloride-stimulated glutamate release in tissue from young (4 month) but not older (22 month) rats, and only in the presence of calcium (“Interleukin-1 beta-STIMULATES-Glutamate” (although the predication itself is wrong)). In a study of the influence of IL-1 beta on memory consolidation, Gonzalez et al. [60] reported that intrahippocampal injection of IL-1 beta in adult male Wistar rats decreases glutamate release from dorsal hippocampus synaptosomes after contextual fear conditioning (“Interleukin-1 beta-INTERACTS_WITH-CRK protein, human”). The studies resulting from this query (including “brain”) and the previous one (not including “brain”) are given in Table 3.

**Table 3:** Summary of articles discussing IL-1 beta and glutamate

*Study*	*Subjects*	*Method*	*Result/Conclusion*
Xiaoqin et al. [54]	Cerebral cortex and hippocampus of rats	Injection of IL-1 beta and IL-6; immuno-histochemistry	Increased glutamate and decreased GABA observed
Donnelly et al. [55]	Synaptosome pellets prepared from brains of 8-12 week-old BALB/c female mice intoxicated with glycerol	In vitro techniques	Report increased IL-1 beta levels and decreased glutamate release in hippocampus tissue
Hu et al. [56]	Human astrocyte cultures subjected to several cytokines and 3H-glutamate	Northern blot analysis and other in vitro techniques	Proinflammatory cytokines inhibit astrocyte glutamate uptake
Huang et al. [57]	Intracerebroventricular injection of IL-1beta in adult male New Zealand white rabbits	Intracerebral microdialysis	Glutamate induced by IL-1 beta
Mascarucci et al. [58]	Intraperitoneal injection of IL-1 beta in freely moving male Sprague–Dawley rats	Brain microdialysis	Increased glutamate released in the nucleus tractus solitarius
Murray et al. [59]	Synaptosomes prepared from male Wistar rats	Immunoblotting with specific antibody	IL-1 beta inhibits potassium chloride-stimulated glutamate release in tissue from young (4 month), in the presence of calcium
Gonzalez et al. [60]	Adult male Wistar rats	Intrahippocampal injection of IL-1 beta; preparation of synaptosomes; in vitro technique to assay glutamate release	IL-1 beta decreases glutamate release from dorsal hippocampus synaptosomes after contextual fear conditioning

The query which retrieved the first two studies used the term “brain”, and that which retrieved the other studies used the term “epilepsy”)

#### Disorder and location not specified

The Semantic MEDLINE query (“interleukin 1” AND glutamate) retrieved 3232 predications from 289 citations. The research we saw in the summarized graph focused on neuronal involvement. For example, the predication “Glutamate-COEXISTS_WITH-Interleukin-1 beta” was extracted from Casamenti et al. [61], which looked at the involvement of inflammation with Alzheimer’s disease. IL-1 beta was injected into the nucleus basalis of adult male Wistar rats. The authors reported a marked increase in glutamate (revealed through microdialysis). Prow and Irani [62] used immunoblotting and immunohistochemistry, cytokine assays, and histological analysis to examine spinal cord tissue extracted from mice challenged with neuroadapted Sindbis virus. Based on analysis of levels of astroglial glutamate transporter (which removes glutamate from the synaptic cleft), IL-1 beta, and glutamate, they claimed that the increase of IL-1 beta in response to the virus disrupts glutamate homeostasis. They concluded (in the abstract) that their data “provide one of the strongest in vivo links between innate immune responses and the development of excitotoxicity demonstrated to date.” (“Interleukin-1 beta-INTERACTS_WITH-Glutamates”).

In a sample of citations from which Semantic MEDLINE did not extract a predication, Fogal et al. [63], for example, investigated the etiology of hypoxic-ischemic brain damage in IL-1R1 null mutant, mGluR1-/-, and wild-type control mice. From both in vitro and in vivo experiments, they concluded that IL-1 beta makes a significant contribution to such neuronal injury and that it increases extracellular glutamate as part of the mechanism. Yan and Weng [64] used both in vitro and in vivo techniques to study the mechanisms by which IL-1 beta interacts with glutamate in neuropathic pain experimentally induced in young adult male Sprague-Dawley rats. They concluded that IL-1 beta uses presynaptic NMDA receptors to enhance glutamate release from primary afferents in neuropathic rats. Yan et al. [65] analyzed tissue extracted from young adult male Sprague-Dawley rats subjected to partial sciatic nerve ligation. Based on several in vitro techniques to determine the mechanisms involved, they concluded that IL-1 beta contributes to neuropathic pain by suppressing glial glutamate uptake. The research reported in this section is summarized in Table 4.

**Table 4:** Summary of articles discussing IL-1 beta and glutamate (disorder and location not specified in query)

*Study*	*Subjects*	*Method*	*Result/Conclusion*
Casamenti et al. [61]	Adult male Wistar rats	IL-1 beta injected into nucleus basalis; microdialysis	Significant increase in glutamate
Prow and Irani [62]	Spinal cord tissue extracted from mice challenged with neuroadapted Sindbis virus	Immunoblotting and immunohistochemistry, cytokine assays, and histological analysis	Increase of IL-1 beta in response to the virus disrupts glutamate homeostasis (development of excitotoxicity)
Fogal et al. [63]	IL-1RI null mutant, mGluR1-/-, and wild-type control mice	Both in vitro and in vivo experiments	IL-1 beta increases extracellular glutamate as part of the mechanism of neuronal injury
Yan and Weng [64]	Young adult male Sprague-Dawley rats	In vitro and in vivo techniques to study experimentally induced neuropathic pain	IL-1 beta enhances glutamate release from primary afferents
Yan et al. [65]	Tissue from young adult male Sprague-Dawley rats subjected to partial sciatic nerve ligation	Several in vitro techniques	IL-1 beta contributes to neuropathic pain by suppressing glial glutamate uptake

After having seen considerable research suggesting that IL-1 beta may increase glutamate, facilitate its receptors, or inhibit its uptake by glial cells in the context of epilepsy and other neuronal disturbances, we turned to glutamate and IBD.

### Glutamate and inflammatory bowel disease

In order to investigate glutamate and gastrointestinal phenomena, we issued two queries to Semantic MEDLINE, one focused on anatomy and another on disease. The anatomy-focused query (glutamate AND (bowel OR colon OR intestine OR gastrointestinal OR stomach)) retrieved 4718 predications from 500 citations. Many of these discuss the relevance of normal levels of glutamate and its receptors to gastrointestinal processes. For example, two recent reviews highlight the prominence of glutamatergic phenomena underpinning the mechanisms of gastrointestinal functions. The predication “Gut-LOCATION_OF-Glutamate” was extracted from Julio-Pieper et al. [66], which states that glutamate is the main neurotransmitter of the brain-gut axis (realized in part by the vagus nerve). (Metabotropic) glutamate receptors occur in the brain as well as throughout the gastrointestinal tract, from the mouth to the large intestine, and are relevant to digestion as a whole. These receptors are involved in several gastrointestinal reflexes, including swallowing, gastric accommodation, and emesis [67].

In one of the citations retrieved with PubMed that did not produce a SemRep predication, Clarke et al. [68] investigated the kynurenine pathway of tryptophan degradation in plasma samples from 10 male patients with irritable bowel syndrome (IBS) and 26 controls. High performance liquid chromatography revealed that concentration of the neuroprotective metabolite kynurenic acid (an antagonist of the NMDA glutamate receptor) was decreased in the IBS subjects.

The disease-focused query (glutamate AND (“inflammatory bowel disease” OR colitis)) retrieved 995 predications from 89 citations. In the summarized graph, “Colitis-PROCESS_OF-Rattus norvegicus” was extracted from Varga et al. [69]. In this study, kynurenic acid, an antagonist of NMDA (a glutamate receptor), was administered to male Wistar rats after inducing colonic inflammation with trinitrobenzene sulfonic acid. Measurements conducted on anesthetized animals as well as on blood samples and colon biopsies indicated a significant modulatory effect, including reduced inflammatory enzyme activities, decreased intestinal motility, and increased tone of the colon.

Several of the MEDLINE citations from which Semantic MEDLINE did not extract a predication report an association between glutamate and intestinal phenomena. For example, Carpanese et al. [70] conducted a study based on in vitro cell cultures from adult male rats. Based on immunocytochemistry, they conclude that blockade of glutamate receptors (NMDA and AMPA/kainite) may protect enteric neurons subjected to in vitro chemically-induced ischemic injury followed by reperfusion.

We then sought additional information on kynurenic acid and its potential role in mitigating gastrointestinal disturbances. The PubMed query, ((“kynurenic acid” OR kynurenine) AND “inflammatory bowel disease”) returned 7 citations. One of these was Forrest et al. [71], in which serum concentrations of purines and kynurenines were measured in patients with mild IBD. In noting increased levels of kynurenic acid compared to controls, they concluded that kynurenine modulation of glutamate receptors is involved in the symptoms of IBD, either as a response to an abnormality or as a primary abnormality itself. The studies we cite on glutamate and IBD are given in Table 5.

**Table 5:** Summary of articles discussing glutamate and inflammatory bowel disease

*Study*	*Subjects*	*Method*	*Result/Conclusion*
Clarke et al. [68]	Male patients with irritable bowel syndrome and healthy controls	High performance liquid chromato-graphy on plasma samples	Kynurenic acid was decreased in patients with disease
Varga et al. [69]	Male Wistar rats after inducing colonic inflammation with TNBS	Measurements on anesthetized animals and on blood samples and colon biopsies after administration of kynurenic acid	Reduced inflammatory enzyme activities, decreased intestinal motility, and increased tone of the colon
Carpanese et al. [70]	Enteric neuron cultures from adult male rats	In vitro ischemic injury; reperfusion; blockage of glutamate receptors; Immuno-cytochemistry to measure cytotoxicity	Blockade of glutamate receptors (NMDA and AMPA/kainite) may be neuroprotective
Forrest et al. [71]	Patients with mild IBD	Measured serum concentrations of purines and kynurenines	Kynurenine modulation of glutamate receptors is involved in the symptoms of IBD

Finally, we issued two PubMed queries, one disease-focused and a second anatomy-focused, to look for research reporting on the interaction of IL-1 beta and glutamate in IBD. The first, (interleukin-1 AND glutamate AND (“inflammatory bowel disease” or colitis)), returned no citations. The second, (interleukin-1 AND glutamate AND (bowel OR colon OR intestine OR gastrointestinal OR stomach)), retrieved three citations, none of which discuss the interaction of IL-1 beta and glutamate in the etiology of gastrointestinal disorders. Saperas et al. [72] discuss a possible effect of IL-1 beta on gastric acid secretion but do not mention the interaction of interleukin-1 beta and glutamate. Morrow et al. [73] investigated the effect of murine IL-1 beta on gastric contractility, but did not address the relation of IL-1 beta and glutamate in IBD. Finally, Qu et al. [74] present a review of epigenetic phenomena in gastric cancer. As part of the discussion, IL-1 beta and glutamate are mentioned, but their interaction in IBD is not addressed.

## Discussion

The iterative approach used during discovery browsing mirrors the iteration observed in studies of complex problem solving [75] and is recognized more generally as an inherent part of the information seeking process [76–78],characterized as six stages: task initiation, selection, exploration, focus formulation, collection, and presentation^1^. This work shows how predicates provided by SemRep and the interactive Semantic MEDLINE interface supports a user as they iterate between selection, exploration, and focus formulation steps.

Based on the results of this study, the statistical correlation found in clinical data [9] can be explained by an increase in glutamate due to IL-1 beta which is involved in the etiology of both IBD and epilepsy. To recapitulate the research that supports that claim, we first looked at IL-1 beta involvement with IBD and epilepsy individually. It is widely accepted that IL-1 beta is etiologically associated with IBD. Regarding epilepsy, there is considerable research suggesting that IL-1 beta is crucially involved in the mechanism of that disorder, and, further, that excess glutamate, being excitotoxic, also contributes to the etiology of epilepsy and seizure.

We next looked at research investigating the interaction of IL-1 beta and glutamate in epilepsy. Several studies based on both animal and human in vitro and in vivo studies suggest that IL-1 beta induces glutamate activity increasing glutamate, facilitating its receptors, and inhibiting its uptake by glial cells, although some studies report that IL-1 beta inhibits glutamate under their research conditions.

**Fig. 3:**
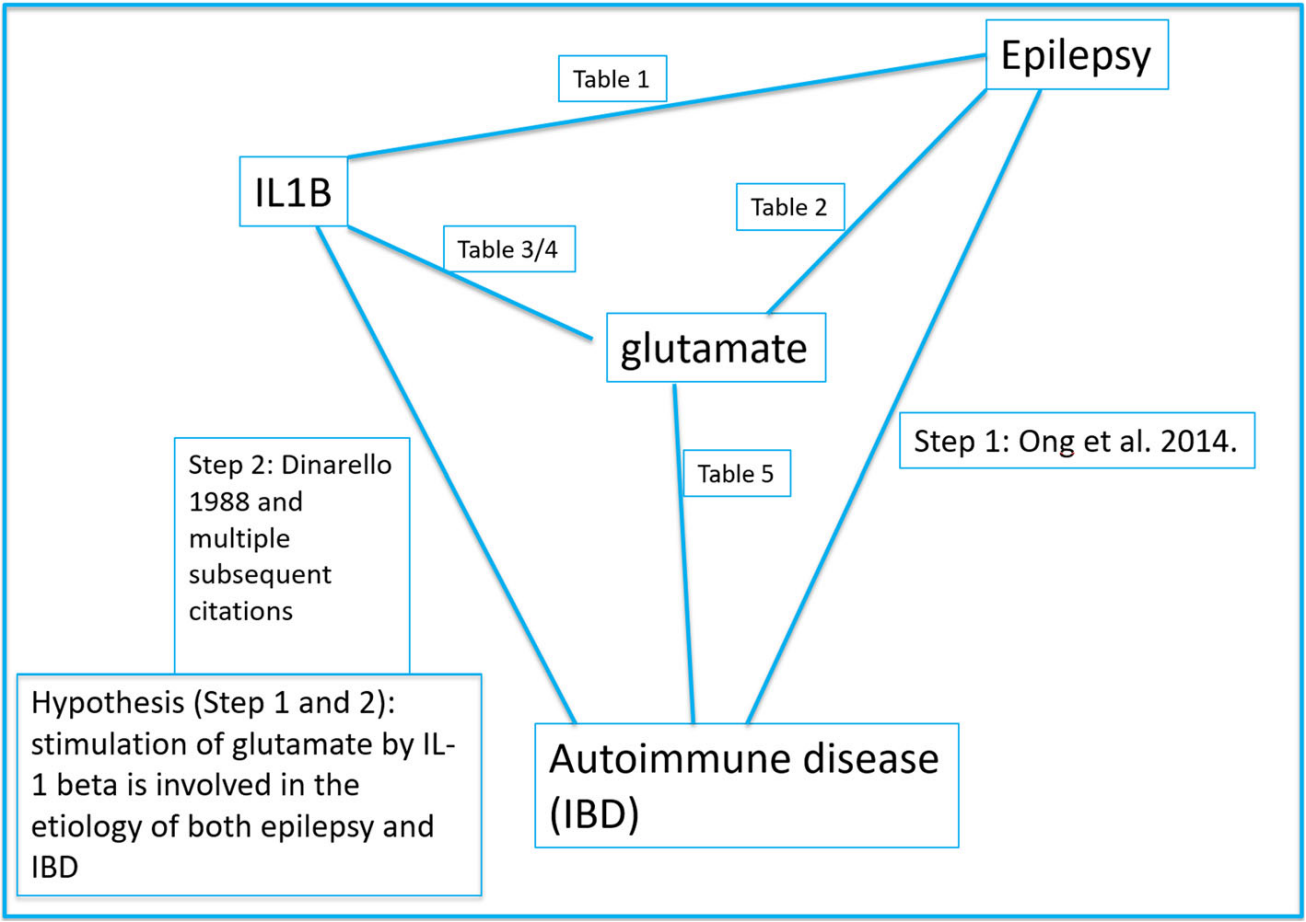
Overview of the hypothesis and supporting research. Increase in glutamate due to IL-1 beta may be involved in the etiology of both IBD and epilepsy

Glutamate plays an important role in several normal gastrointestinal functions, and some research suggests that excessive levels of glutamate contribute to disturbances. The strongest evidence for this is that the NMDA antagonist kynurenic acid has demonstrated therapeutic value in IBD models. Although it is widely accepted that IL-1 beta plays a crucial role in IBD, we did not find any studies investigating the interaction of IL-1 beta and glutamate in IBD. In the context of the rest of our findings, this would seem to be a potentially valuable direction to pursue. Figure 3 shows an overview of the hypothesis and supporting research.

Finally, we queried PubMed in an effort to determine whether the hypothesis that elevated glutamate levels due to IL-1 beta are part of the mechanism of both IBD and epilepsy is novel. Despite this supporting evidence, the PubMed query, (“interleukin 1” AND glutamate AND (“inflammatory bowel disease” OR colitis) AND (seizure OR epilepsy)) returned no citations, indicating the novelty of this hypothesis.

There are some limitations to our study. First, discovery browsing is a means of generating hypotheses, not of determining evidence. Therefore, hypotheses derived must be subjected to experimental investigation to determine their value and significance. Secondly, SemRep is not perfectly accurate; its precision is estimated to be about 75% (lower for predications involving cellular/molecular interactions) and its recall is lower (estimated to be approximately 50% [25]). Note, however, that automatic summarization filters out some of the precision errors, and also that a precision error can still be useful in pinpointing MEDLINE citations that merit closer scrutiny (as shown in one of the examples above). On the other hand, by issuing queries directly to PubMed, in addition to Semantic MEDLINE, we aimed to mitigate the effect of recall errors. Lastly, our methodology relies solely on semantic predications and manual inspection of MEDLINE citations that they are extracted from. There is a wealth of taxonomic and relational knowledge which can be mined directly from UMLS and biomedical ontologies (e.g., Gene Ontology) and incorporated into discovery browsing to pinpoint other interesting associations. We plan to explore this integration in future work.

## Conclusion

Data-mining studies using population-scale data have the potential to identify novel correlations; however, they generally do not provide plausible explanations for these correlations. Although there is no single experiment that demonstrates the direct connection between interleukin and glutamate and inflammatory bowel disease or colitis, the discovery browsing approach used in this paper demonstrates that there is evidence available to support the mechanistic connection between the observation made in a population study [9] that epilepsy and IBD often co-occur and that inflammation is likely involved. We followed cooperative reciprocity in the discovery browsing methodology, which involves a complementary interaction between the user and Semantic MEDLINE and PubMed, with the former suggesting ideas to pursue and the latter two providing support or disconfirmation. Based on the results of this method, we proposed the hypothesis that IL-1 beta influence on glutamate levels is involved in the etiology of both epilepsy and IBD. This hypothesis can underpin the development of more effective therapeutic approaches for both epilepsy and IBD.

We conclude by observing that semantics-based discovery browsing is complementary to population-based correlation studies. The former provides depth (mechanism), while the latter provide breadth. It is possible to start with either and use the other for support. In this study we started with a correlation-based study and used discovery browsing to elucidate a mechanism. Hypotheses suggested by discovery browsing could also be supported with population studies.

## Abbreviations

EHR: Electronic health record IBD: Inflammatory bowel disease IBS: Irritable bowel syndrome IL: Interleukin IL-1R1: Interleukin-1 receptor type 1 LBD: Literature-based discovery OVLT: OrganumRrganum vasculosum laminae terminalis UMLS: Unified medical language system

## Footnotes

1 Prior work that studied biomedical researchers as they systematically reviewed the literature also found that this process holds, with one additional step (synthesis) that occurs between the collection and presentation stages [79, 80].
